# Imaging and safety profiling of inhaled siRNA RyR2 in human respiratory models

**DOI:** 10.1007/s00204-026-04370-7

**Published:** 2026-04-15

**Authors:** Valeria Bettinsoli, Magdalena Erlacher, Valentina Galbiati, Marina Marinovich, Emanuela Corsini, Doris Wilflingseder

**Affiliations:** 1 Department of Pharmacological and Biomolecular Science (DiSFeB) “Rodolfo Paoletti”, Università degli Studi di Milano, Via Giuseppe Balzaretti 9, 20133 Milan, Italy; 2 Department of Pharmacy, Università degli Studi di Napoli Federico II, Via Domenico Montesano 49, 80131 Napoli, Italy; 3 Ignaz Semmelweis Institute, Interuniversity Institute for Infection Research, Vetmeduni, Vienna, Austria

**Keywords:** New approach methodologies, NHBE, 3D model, Nucleic acid drugs, Immunotoxicology, RyR2, Nanoparticles

## Abstract

RNA therapeutics hold strong potential for treating genetic disease, yet progress is often limited by delivery, stability, and safety concerns. Here, we profile respiratory safety and cellular uptake of an inhaled siRNA targeting the cardiac RyR2 gene, which is responsible for catecholaminergic polymorphic ventricular tachycardia (CPVT), delivered via inhalation with calcium phosphate nanoparticles (CaP NPs). Barrier integrity, cytotoxicity, siRNA uptake, and immune activation, were assessed using a human 3D normal bronchial epithelial model (NHBE) under Air–Liquid Interphase (ALI) and dendritic cells (DCs), tested in monoculture and in co-culture. Barrier function, measured by transepithelial electrical resistance (TEER), remained stable after 48 h of exposure to 400 nM siRNA RyR2, scramble control, or CaP NPs, indicating preserved epithelial performance. Confocal imaging showed efficient internalization of Cy5-labeled siRNA in both mono- and co-cultures. Cytokine profiling revealed IL-8 release across all conditions in NHBE and NHBE + DC models, with IL-6 and TNF-α limited to immune-competent co-cultures; IFN-γ was below the limit of detection. No cytotoxicity was observed. Together, these data demonstrate that CaP NP–mediated delivery achieves robust siRNA uptake without compromising airway barrier integrity, while eliciting only modest, context-dependent immune responses (primarily IL-8 and TNF-α in the presence of DCs). This work supports the respiratory safety and translational potential of inhaled RyR2-targeting siRNA for CPVT.

## Introduction

RNA-based approaches are particularly advantageous for modulating gene expression or producing therapeutic proteins, making them well-suited for diseases with clearly defined genetic targets (Bettinsoli et al. 2025). Despite significant progress and the approval of several RNA-based therapeutics, significant challenges remain in their development. Key strategies, such as enhancing intracellular RNA delivery and improving metabolic stability, are actively pursued to address these limitations (Zhu et al. 2022). Current research emphasizes optimizing gene silencing by designing chemically modified siRNA molecules while minimizing adverse in vivo effects (Alshaer et al. 2021). Safety evaluation of RNA-based drugs is a critical aspect of their development and achieving high target specificity is essential to avoid unintended off-target effects, which can arise from both sequence-dependent and sequence-independent mechanisms (Yu et al. 2020). Watson–Crick base pairing and RNA hybridization are responsible for both on-target effects that may manifest as exaggerated pharmacological responses or as harmful effects in non-target organs, and off-target effects related to oligonucleotide activity on transcripts other than the intended target (Arechavala-Gomeza and Garanto 2022). In the context of the off- target effects, immune-related toxicity represents another major challenge, often contributing to dose-limiting adverse effects and clinical trial failures (Beg et al. 2017). Endosome or membrane Toll-Like Receptors (TLRs), as well as cytosolic sensors such as RNA-dependent protein kinase activation, are typically involved in this toxicity (Anderson et al. 2010). Preclinical safety assessments, including human in vitro and ex vivo assays, are essential for identifying formulations that combine potency with tolerability. These human cell-based models provide cost-effective tools for evaluating pharmacological activity, cytotoxicity, and species-specific immune responses, thereby guiding the refinement of RNA drug candidates (Bitounis et al. 2024).

siRNA RyR2 was developed within the Italian National Center for Gene Therapy and RNA-Based Drug Technology program, and it was designed to selectively target the mutated RyR2 sequence, causing dominant catecholaminergic polymorphic ventricular tachycardia (CPVT). Inhalation is the route of administration of siRNA RyR2, and this route of delivery for targeting cardiac tissue is supported by experimental evidence (Mills et al. 2009; Savi et al. 2014). In vivo studies have confirmed that inhalable Calcium Phosphate Nanoparticles (CaP NPs) carrying therapeutic biomolecules can migrate from the lungs to the heart, are able to enter cardiomyocytes through endocytosis, and to release their cargo via acidic dissolution within endosomes (endosomal escape), thereby enabling treatment of cardiovascular diseases (Di Mauro et al. 2016; Greeley 2017; Janas et al. 2018).

It is well known and reported that both innate and acquired immune systems can be activated by external substances such as particulates, gases, pathogens, and toxins via the inhalation route of exposure (Greeley 2017). The activation of the respiratory system by these agents can trigger cytokine release, which in turn promotes the activation of both type 1 and 2 lymphocytes that results in their elimination (Xu et al. 2016).

This study was set out to evaluate the respiratory safety of an inhaled RyR2-targeting siRNA using a 3D in vitro model. A human 3D airway epithelial model, tested both as a monoculture and in co-culture with dendritic cells, was used. We evaluated potential off-target and immunotoxic effects of this RNA therapeutic. The approach establishes a versatile preclinical framework for safety assessment of inhaled RNA-based therapies.

## Materials and methods

### Chemicals

Fetal Bovine Serum (FBS) was purchased by Capricorn Scientific, (Ebsdorfergrund, Germany). Dulbecco’s Phosphate Buffered Saline (PBS), RPMI-1640, Penicilin Streptomicin 10 mg/ml were purchased by Sigma Aldrich (St. Louis, MO, USA). siRNA RyR2 sense strand: UAUUUUGCUUG-CAACUUUUAC [dT][dT], antisense strand: GUAAAAGUUGCAAGCAAAAUA [dT][dT]; siRNA scramble: MISSION® siRNA Universal Neg. Controls were purchased by Merck (Darmstadt, Germany). RNAse-free ultrapure water, GlutaMAX Supplement (100x), Carboxyfluorescein succinimidyl ester (CFSE) and CyQUANT^TM^ LDH Cytotoxicity Assay Kit were purchased by Thermo Fisher Scientific (Waltham, Massachusetts, USA) PneumaCult -Ex Plus Basa Medium, PneumaCult-Ex Plus 50x Supplement, PneumaCult ALI Basal Medium medium, PneumaCult-Ex Plus 10x Supplement, PneumaCult-ALI Maintenance supplement and Hoechst 20 mM were purchased by Stemcell (Vancouver, British Columbia, Canada); Ficoll-PaqueTM Premium was purchased by Cytiva (Marlborough, Massachusetts, USA); recombinant Human GM-CSF (100 ng/ml) was purchased by Biolegend (San Diego, California, Stati Uniti); Human IL-4 (35 ng/mL) was purchased by Milteny Biotec (Berish Gladbach, Germany); Fixation Buffer, 1x Intracellular Staining Permeabilization Wash Buffer 10X and BD IMag^TM^ Anti-Human CD14 Magnetic Particles—DM were purchased by BD Bioscence (Franklin Lakes, New Jersey, USA). Pan Cytokeratin Monoclonal Antibody (AE1/AE3), Alexa Fluor™ 488, was purchased by eBioscience™ (Fisher Scientific, Waltham, Massachusetts, USA). Phalloidin-iFluor 555 Reagent was purchased by Abcam (Cambridge, UK). Heparin 0.2% was purchased by SciencCell (Carlsbad, California, USA).

### Cell cultures

Normal Human Bronchial Epithelial Cells (NHBE, Lonza, Basilea, Switzerland) were seeded in a 0.4 µm polyester membrane insert (Costar, Corning, New York, USA) at the concentration of 1 × 10^5^ cells/transwell. In the basal compartment, 700 µL of growth medium PneumaCult -Ex Plus Basal Medium (Stemcell) supplemented with PneumaCult-Ex Plus 50 × Supplement (Stemcell) and hydrocortisone 0.48 µg/mL (Stemcell,) were added.

Classical CD14^+^ monocytes were isolated from human donors (reg. no. 2024027) and differentiated to obtained dendritic cells (DC). PBMCs were first obtained by Ficoll gradient centrifugation using Ficoll-PaqueTM Premium (Cytiva). The isolation of monocytes was performed with BD IMag™ Anti-Human CD14 Magnetic. Isolated monocytes were resuspended in RPMI-1640 medium (Sigma Aldrich), supplemented with 1% of GlutaMAX Supplement (100x) (Thermo Fisher Scientific), 1% of Penicilin/Streptomicin 10 mg/mL (Sigma Aldrich), and 10% of Fetal Bovine Serum (FBS) (Capricorn Scientific). Recombinant Human GM-CSF (100 ng/ml) (Biolegend) and Human IL-4 (35 ng/mL) (Miltenyi) were added to induce monocytes differentiation into DC. Cells were seeded at a density of 10^6^ cells/mL into five wells of a 6-well plate, with 3 mL of suspension per well. Cultures were maintained at 37 °C in a humidified incubator with 5% CO_2_ for 5 days to allow monocyte differentiation into DCs.

### Preparation of air–liquid interface (ALI) culture

NHBE cells grew in submerged conditions for 3–4 days, time needed to reach near- complete confluence. After that time, the cells were transferred to Air Liquid Interface (ALI) conditions (Day 0) and maintained at 37 °C in humified atmosphere with 5% of CO_2_. 700 µl of medium in the basal compartment were changed 3 times a week using PneumaCult ALI Basal Medium (Stemcell) with PneumaCult-Ex Plus 10x Supplement (Stemcell), PneumaCult-ALI Maintenance supplement (100x) (Stemcell), heparin 4 µg/mL (SciencCell), hydrocortisone (Stemcell) 0.48 µg/mL (Stemcell), posaconazole 1.9 µg/mL (SigmaAldrich), Penicillin/Streptomycin 0.1 mg/mL (Sigma Aldrich).

2 × 10^5^ DCs/well were added on the basolateral side of fully differentiated cells (> 28 days). DCs were harvested from the 6-well plate, counted and resuspended to obtain the final concentration of 4 × 10^6^ cells/mL. For confocal microscopy visualization, DCs were labeled with CFSE prior to co-culture. Cells at a concentration of 4 × 10^6^ cells/mL were resuspended in PBS and incubated with 5 µM CFSE for 20 min at 37 °C in a humidified 5% CO_2_ incubator.

### Transepithelial electrical resistance (TEER) measurement

After four weeks, the epithelium was fully differentiated, transepithelial electrical resistance (TEER) was measured using a Millicell® ERS 3.0 Digital Voltohmmeter (Merck, Darmstadt, Germany). Prior to measurement, 150 µL of medium were added to the apical side of the transwell, and cells were allowed to equilibrate for 2 min. Following TEER measurement, the medium was rapidly removed. Reported TEER values were corrected for the resistance and surface area of the transwell filters.

### Exposure to siRNA RyR2, scramble control and CaP NP

The basal compartment was exposed to siRNA RyR2, its scramble control and CaP NPs at a final concentration of 400 nM. At this concentration, the amount of CaP NP tested is 0.108 mg/mL. The selected concentration fourfold higher than the in vitro therapeutic dose (100 nM) (Bettinsoli et al. 2025). For cell imaging, Cy5-labeled siRNA RyR2 and scrambled control were used at the same concentration (400 nM). Cell culture was maintained at 37 °C in a humified atmosphere with 5% of CO_2_ for 48 h. Following the exposure time, TEER measurement was performed as previously described. Basal compartment medium was collected and stored at − 20 °C. Confocal staining imaging was performed on the epithelium to visualize the drug within the tissue.

### Cytotoxicity evaluation after siRNA RyR2, scramble control and CaP NP exposure in co- culture system

Cytotoxicity was assessed after 48 h of exposure of NHBE co-cultured with DC by measuring the leakage of lactate dehydrogenase (LDH) using the CyQUAN^TM^ LDH Cytotoxicity Assay Kit (Thermo Fisher) following the manufacturer’s instructions. Absorbance was measured at 680 nm and 490 nm using a spectrophotometer. Results are expressed as percentage as LDH release normalized on naïve cells.

### Cell morphology and siRNA Cy5 labelled localization

Confocal microscopy allows the visualization and the localization of the siRNA and scramble Cy5-labeled in the epithelium of the mono and co- culture. After 48 h of exposure, cells were fixed with Fixation Buffer (Cytofix, BD BioscenceBioscience, Franklin) and the intracellular immunofluorescent staining was performed using 1x Intracellular Staining Permeabilization Wash Buffer 10X (BD Bioscience). Antibodies to detect nuclei (Hoechst 33342), cytokeratin (Pan Cytokeratin Monoclonal Antibody AE1/AE3), Alexa Fluor^TM^ 488 (eBioscience^TM^) for monoculture and F-actin (Phalloidin, Phalloidin-iFluor 555 Reagent, Abcam) for co-culture. After staining, the epithelium was mounted in Mowiol and the sample was imaged with Operetta CLS System using the 63x objective in confocal mode (RRID:SCR_018810, Revvity, formerly PerkinElmer). Analysis was performed using the Harmony software 4.9

### Cytokines release induced by siRNA RyR2, scramble control and CaP NP

NHBE cells were treated with siRNA RyR2, scramble control and CaP NPs formulations for 48 h. Supernatants were collected for cytokine measurement and stored at − 20 °C until measurement. Cytokine production including Interleukin (IL)−8, IL-6, Tumor Necrosis Factor (TNF)-α and Interferon (IFN)-γ was assessed in cell-free supernatants by specific sandwich enzyme-linked immunosorbent assays (ELISA) that are commercially available (BD biotechne). Results were expressed as fold-change of released cytokines of treated samples versus control cells (Naïve). Sensitivity range: IL-6 9.38–600 pg/mL; IFN-γ 9.38–600 pg/mL; TNFα 15.6–1000 pg/mL; IL-8 8–500 pg/mL.

### Statistical analysis

All in vitro experiments were performed at least three times (n = 3). Statistical analysis was performed using GraphPad version 9.5.0 for MacOS (GraphPad Software, San Diego, CA, USA). ANOVA was used to estimate the effects of chemicals, time, and doses and to compare the between-group differences. 2Way ANOVA Šídák’s multiple comparisons test was used for comparison of the TEER measurement before and after treatment. Paired t-test and Wilcoxon test were used for cytokine release analysis. Kolgomorov-Smirnov and Shapiro–Wilk tests were used as normality tests. Differences were considered significant at *p* ≤ 0.05 (*).

## Results

### Preserved airway barrier integrity after 48 h exposure to siRNA RyR2

**Fig. 1:**
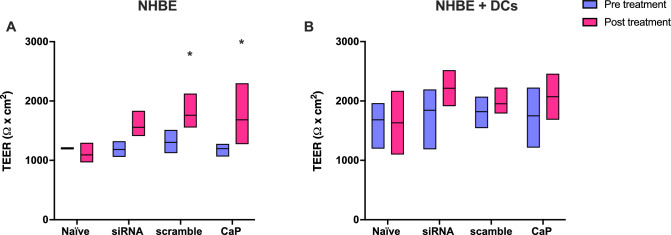
TEER measurement of NHBE (**A**) and NHBE + DCs (**B**) after 48 h of exposure to siRNA RyR2, scramble control, and CaP NPs at the concentration of 400 nM. Results are expressed as Ω × cm^2^and represented as mean ± SEM (n = 3). Statistical analysis was performed by two-way ANOVA, Šídák's multiple comparisons test with *p < 0.05 vs naïve cells post treatment

To confirm tight barrier formation of the model, transepithelial electrical resistance (TEER) was measured in NHBE monocultures and NHBE + DC co-cultures after 28 days of ALI culture. As shown in Fig. 1, TEER values indicated well-formed tight junctions, validating the model for drug exposure. The TEER values of NHBE + DC co-cultures, both before and after treatment, were higher compared to the NHBE monocultures. After 48 h of treatment with 400 nM siRNA RyR2, scramble control, or CaP NPs, TEER remained stable or showed an increase to approximately 1500 Ω·cm^2^, indicating preserved epithelial integrity. The no effect on TEER is also paralleled by the absence of cytotoxicity as assessed by LDH leakage (data not shown). These findings suggest that neither the siRNA nor the CaP NPs carrier disrupted tight junctions, reducing concerns about paracellular leakage and supporting the compatibility of CaP NP–siRNA formulations with human airway epithelia.

### Confocal imaging reveals internalization of siRNA RyR2 Cy5 labelled

**Fig. 2:**
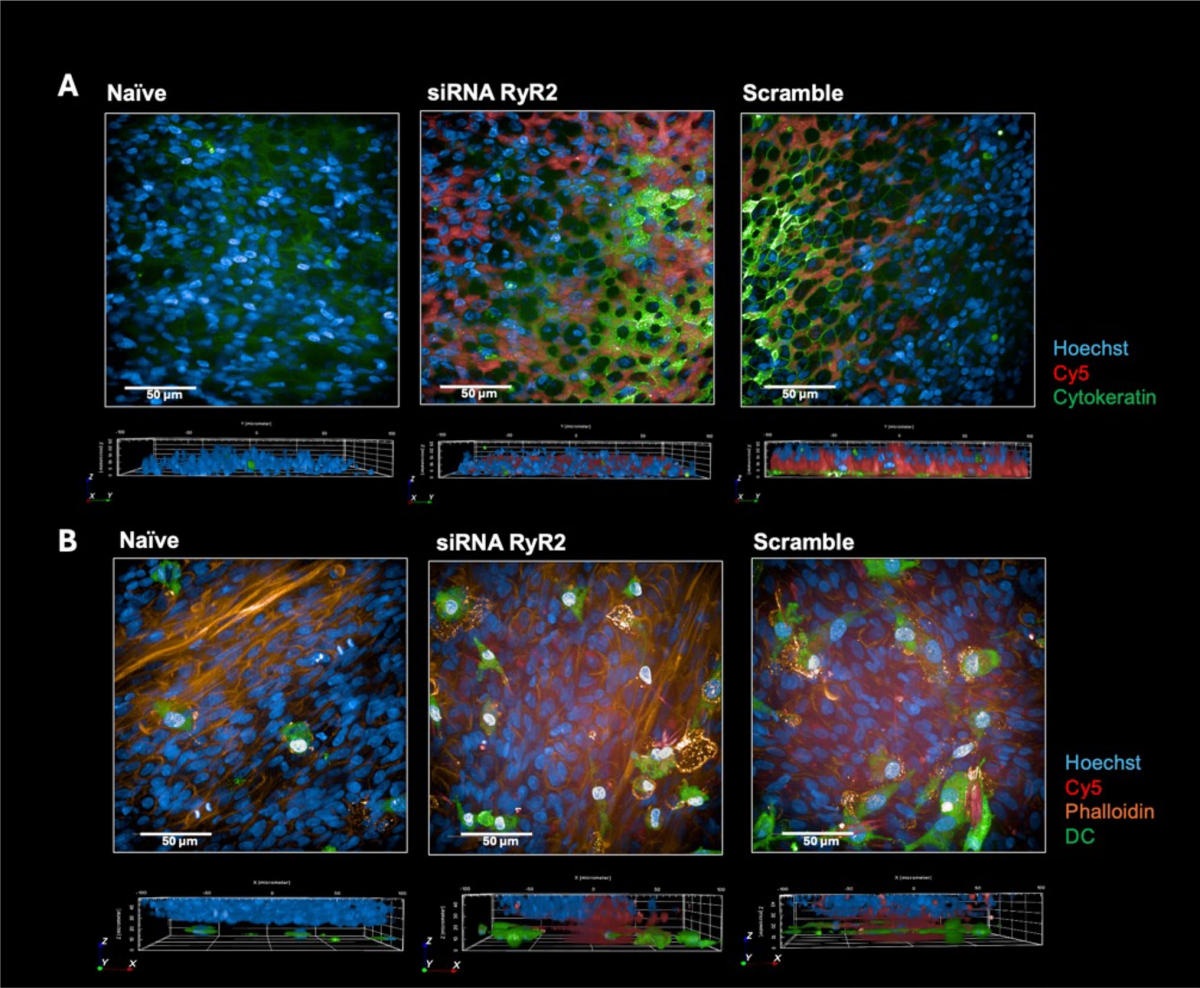
Internalization of Cy5-labeled siRNA RyR2 and scramble control in 3D pseudostratified epithelial mono-culture (Panel **A**) and co-culture (Panel **B**). Representative images include a packed 3D stack and a 3D reconstruction, showing merged color channels for the three conditions: naïve, siRNA RyR2, and scramble control. Images were acquired using the Operetta CLS High-Content Screening system with a 63 × water-immersion objective. Nuclei were stained with Hoechst (blue), cytokeratin (green) for NHBE, actin with phalloidin (orange), and CFSE for DCs (green) for NHBE + DC co-cultures. Internalization of both siRNA and scramble was observed in both NHBE and NHBE + DC cultures Scale bars = 50 µm. Data are representative of three independent experiments from distinct culture areas, averaging ~ 430 cells per condition

To evaluate the uptake of siRNA RyR2 and its scramble control in fully differentiated pseudostratified epithelia, cultures were exposed to Cy5-labeled siRNA for 48 h, then fixed for immunofluorescence. Nuclei were counterstained with Hoechst. In monocultures, epithelial cells were labeled with Alexa Fluor 488–conjugated anti-cytokeratin (Fig. 2A). In co-cultures, dendritic cells were pre-labeled with CFSE, so Alexa Fluor 555–phalloidin was used to visualize epithelial F-actin and avoid spectral overlap (Fig. 2B). Representative images include a packed 3D stack (Fig. 2A, B, upper panels) and a 3D reconstruction (Fig. 2A, B, lower panels). Confocal imaging revealed marked Cy5-siRNA internalization in both mono- and co-cultures, indicating efficient delivery (Fig. 2A, B). Collectively, these results show that CaP NP mediated delivery supports intracellular siRNA uptake under ALI conditions without compromising epithelial integrity.

### siRNA exposure triggers sequence-independent inflammation

**Fig. 3:**
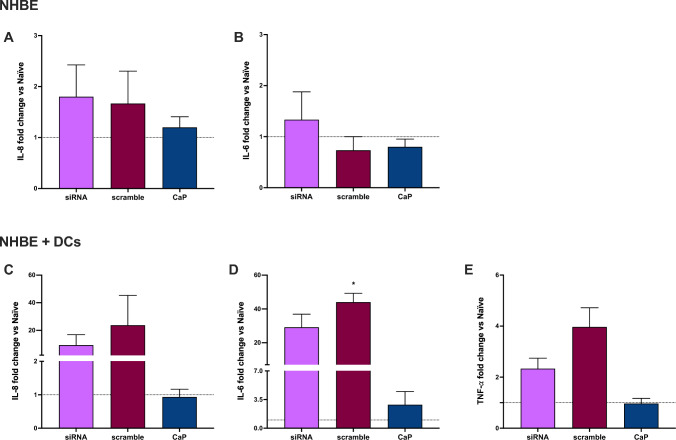
Cytokine release induced by siRNA RyR2, scramble control and CaP NP. NHBE and NHBE + DC were exposed to siRNA RyR2, scramble control and CaP NP at the concentration of 400 nM for 48 h. IL-8 (**A**–**C**), IL-6 (**B**–**D**), TNF-α (**E**) release were assessed by specific ELISA. Results are normalized on the cytokine release in naïve cells (dotted line). Each column represents the mean ± SEM (n = 3). Statistical analysis was performed by One Way Anova, with *p < 0.05 vs naïve cells

After 48 h of exposure, pro-inflammatory cytokines (IL-8, IL-6, TNF-α) were quantified by ELISA. Figure 3 presents responses to siRNA RyR2, scramble control, and CaP NPs as -fold change relative to naïve controls in both, mono- and co-culture settings. A non-statistically significant increase in IL-8 secretion following exposure to both siRNA RyR2 and scramble increased IL-8 secretion in monocultures (Fig. 3A) and co-cultures (Fig. 3C) was observed, with stronger effects in NHBE + DCs (Fig. 3C). IL-6 was statistically elevated only in scramble-treated co-cultures (Fig. 3D) but not in monocultures (Fig. 3B), and TNF-α was detected exclusively in NHBE + DCs (Fig. 3E) with a non-statistically significant increase. IFN-γ remained undetectable across all conditions. The effect on cytokine secretion was stronger for the scramble compared to siRNA RyR2, suggesting that immune activation is partially sequence-dependent, likely reflecting RNA chemistry. The delivery system alone has no effect across all conditions.

## Discussion

This study aimed to investigate the potential respiratory toxicity induced by a specific RNA-based drug, siRNA RyR2, developed to be delivered through the inhalation route. This study examined the effects on epithelial barrier integrity, uptake, and immune activation in differentiated NHBE monocultures and NHBE + DC co-cultures. This study was conducted as a preliminary step in the preclinical assessment of the safety profile of RNA-based drugs delivery via inhalation. The results demonstrate efficient siRNA internalization without compromising barrier function or inducing cytotoxicity but reveal measurable, but not statistically significant for siRNA RyR2, innate immune activation, which has important implications for the design and optimization of inhaled siRNA therapeutics. The delivery system itself has no effect.

Delivery systems are central in RNA drug development. Naked RNAs are highly vulnerable to enzymatic degradation by nucleases and hydrolases and are rapidly cleared by renal filtration (Janas et al. 2018). To address these challenges, RNA therapeutics relies on specialized carriers that protect RNA integrity and enable targeted delivery. However, carrier safety must also be rigorously assessed, as degradation products, infusion site reactions, or unintended tissue accumulation can pose additional risks (Janas et al. 2018). CaP NPs is the encapsulation designed for delivery of the siRNA RyR2 to the heart. This efficient tool allows the drug to be internalized into the cells and to reach the cardiomyocytes with therapeutic effects (Bongianino et al. 2017; Miragoli et al. 2018). As previously mentioned, the siRNA RyR2, is designed to target the heart via inhalation (Degli Esposti et al. 2018; Di Mauro et al. 2016; Xu et al. 2016). The RyR2 sequence encodes a protein that assembles into a homotetrameric ion channel located in the membrane of the junctional sarcoplasmic reticulum. RyR2 channels are spatially aligned with L-type Ca^2^⁺ channels (LTCCs) situated in specialized membrane invaginations called transverse tubules (Bettinsoli et al. 2025). Calcium entry through LTCCs triggers RyR2-dependent Ca^2^⁺ release from the sarcoplasmic reticulum, a mechanism termed calcium-induced calcium release that enables cardiac muscle contraction (Bers 2002). Under physiological conditions, sympathetic stimulation leads to phosphorylation of RyR2, thereby increasing Ca^2^⁺ release from the sarcoplasmic reticulum in myocytes. In contrast, when mutations are present, calcium is inappropriately released from the sarcoplasmic reticulum, resulting in arrhythmic heart activity that can lead to cardiac (Priori et al. 2001). Once in the circulatory system, nanoparticles (NPs) are frequently identified as non-self-entities, leading immune cells to act against them and facilitate their clearance (Lee et al. 2023). Evaluating these interactions is therefore fundamental to achieving safe and efficient delivery strategies (Najahi-Missaoui et al 2020). The immune cell activation, and adaptive immune response, that can result in cytokine release syndrome, are influenced by RNA size, sequence, and the administered dose (Janas et al. 2018). Even if not reaching statistically significance, o with the exception of scramble-induced IL-6 release, our study observed that both siRNA RyR2 and its scramble control induced IL‑8 secretion in NHBE cultures, particularly when co-cultured with DC, and elicited IL‑6 and TNF‑α only in immune‑competent conditions aligns closely with prior reports on TLR‑mediated innate responses in airway epithelium (Kaushal 2023; Koff et al. 2008; Meng and Lu 2017). These signaling pathways are preferentially amplified in co‑cultures that include antigen‑presenting cells, matching our finding that TNF‑α was only detectable when NHBE cells were co-cultured with DCs. Strategies to mitigate off-target risks include designing optimal RNA sequences, incorporating chemical or natural modifications to reduce immunogenicity, and balancing efficacy with safety through precise dosing (Yu et al. 2020). Consequently, the use of suitable in vitro and in vivo models during drug development is critical for reliably assessing both the therapeutic efficacy and the potential toxicity of these systems.

While CaP NP are generally considered biocompatible, recent reviews note their dual role in facilitating cargo delivery while also enhancing immunogenicity when used in vaccine applications (Sun et al. 2021). Interestingly, our data suggest that NP structure alone does not trigger cytokine release, given similar responses for both scramble and targeting siRNA, pointing to siRNA duplex structure as the principal signal.

The TEER values after 48 h of exposure showed a slight increase to around 1500 Ω × cm2, indicating the barrier function in NHBE cells remained upon exposure in both mono and co-culture, these results correlated with an increased phalloidin signal detected by confocal microscopy, suggesting a strengthening of actin filament structure in both siRNA and scramble in NHBE cells. This evidence is supported also from Bartman et al., that following passive siRNA targeting CHD1 and EpCAM transfection NHBEs showed increased TEER. (Bartman et al. 2021).

TNF-α’s presence only in co-cultures underscores the amplifying role of immune cells, consistent with pattern recognition receptor-mediated sensing such as TLR3 or TLR7/8.

These results could suggest that the model composed by NHBE and DC is more sensible to the siRNA drug-induced secretion of inflammatory mediators compared to just NHBE cells.

For inhaled siRNA delivery, maintaining epithelial integrity while ensuring uptake is critical. Our data show that CaP NP–mediated delivery achieves these goals without cytotoxicity, but the identification of a possible innate immune activation, particularly in immune-competent settings, requires mitigation to avoid exacerbating airway inflammation. Early integration of immune-inclusive models and immunotoxicity screening will streamline candidate selection and reduce clinical risk. This study assessed a single dose and time point, with cytokine analysis limited to selected markers, and broader profiling along with direct RyR2 knockdown confirmation are needed to fully interpret biological effects.

In conclusion, siRNA RyR2 and scramble delivered via CaP NPs are efficiently internalized and preserve barrier integrity without cytotoxicity, but induce mild innate immune responses, mainly IL-8 and TNF-α in co-cultures. These effects are likely reflecting siRNA sensing rather than target silencing. Optimizing siRNA chemistry, carrier properties, and dosing strategies will be essential to balance efficacy with minimal off target immunostimulation.

## Data Availability

All data generated or analyzed during this study are included in this published article. Raw data underlying the figures and analyses are available from the corresponding author on reasonable request.
